# Supplementary material for: Inertial Motion Capture-Based Estimation of L5/S1 Moments during Manual Materials Handling
Source: Sensors (Basel). 2022 Aug 26;22(17):6454. doi: 10.3390/s22176454 (PMC9459798; doi:10.3390/s22176454)
Supplement: Supplementary file 1 [file sensors-22-06454-s001.zip › sensors-1854277-supplementary.pdf]

Table S1. Description of MMH tasks configuration and experimental conditions

Trial	Pace	Di	Locations	Load	Rep	Instructions
1	Free	1.5	D – D	Box-10	4	No pre- or post-handling
2	Free	1.5	D – D	Box-10	4	Pre- and post-handling to move the load closer
3	Free	1.5	D – D	Box-10	4	Counterbalance with one leg for lifting and de-posit
4	Free	1.5	L – L	HBox-10	4	-
5	Free	1.5	L – L	LBox-10	4	-
6	Free	1.5	L – L	Bag-20	4	Pre- and post-handling
7	Free	1.5	L – H	Box-10	8	Keep the same grip during the entire transfer
8	Free	1.5	L – M	Box-10	4	Make a stoop for lifting and deposit
9	Free	1.5	L – M	Box-10	4	Simulate a fragile object
10	Free	1.5	L – M	Box-20	4	-
11	Free	1.5	M – M	Box-2	4	Keep the load away from self
12	Free	1.5	M – M	Box-2	4	Keep the load close from self
13	Free	0.75	L – M	Box-20	8	-
14	Free	0.75	L – M	Box-2	8	-
15	Free	0.75	M – H	Box-10	8	Use the potential energy of the load to reach the deposit location
16	Free	0.75	L – H	Box-10	8	Use the potential energy of the load to reach the deposit location
17	Free	0.75	L – L	HBox-10	4	No pre- or post-handling
18	Free	0.75	L – L	HBox-10	4	Pre- and post-handling
19	Free	0.75	L – L	Box-10	4	Grip the load in the width's direction
20	Fast	0.75	L – L	Box-10	4	Fix the feet and prioritize the back twisting
21	Fast	0.75	L – L	Box-10	4	Fix the feet and avoid the back twisting
22	Fast	0.75	L – L	Box-10	4	Fix the feet and delay the weight transfer
23	Fast	0.75	L – L	Box-10	4	Fix the feet and anticipate the weight transfer
24	Fast	0.75	L – M	Box-10	8	Use the potential energy of the load to reach the deposit location
25	Fast	0.75	L – M	Box-2	8	-
26	Fast	0.75	L – M	Box-2	8	Linear and constant speed transfer of the load
27	Fast	1.5	L – M	Box-10	4	Move around and avoid obstacles on the ground
28	Fast	1.5	L – L	Box-10	4	Release the load towards the deposit location
29	Fast	1.5	L – L	Box-20	4	Make a squat for lifting and deposit
30	Fast	1.5	L – L	Box-10	4	Place the feet towards the deposit location when lifting
31	Fast	1.5	L – L	Bag-20	4	No pre-handling

"Box-2" refers to the 2 kg-box with handles; "Box-10" refers to the 10-kg box without handles; "Box-20" refers to the 20-kg box without handles; "LBox-10" refers to the large 10-kg box with handles; "Hbox-10" refers to the 10-kg box with handles; "Bag-20" refers to the 20-kg gravel bag.

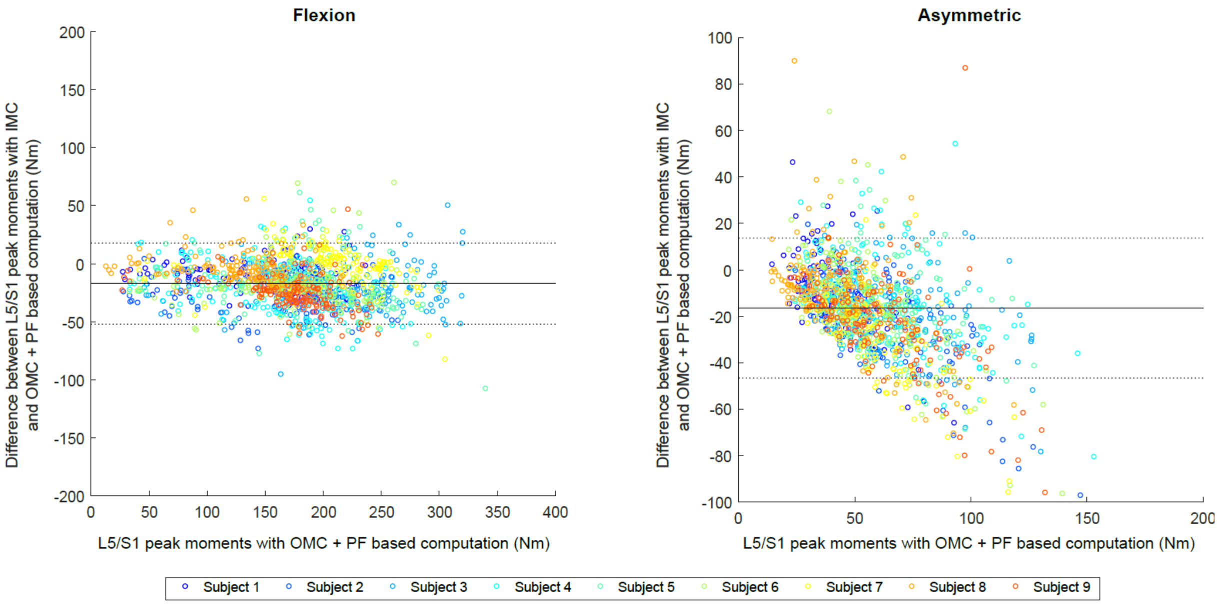

Figure S1. Bland-Altman plot between OMC + PF based and IMC based computations of L5/S1 peak moments.

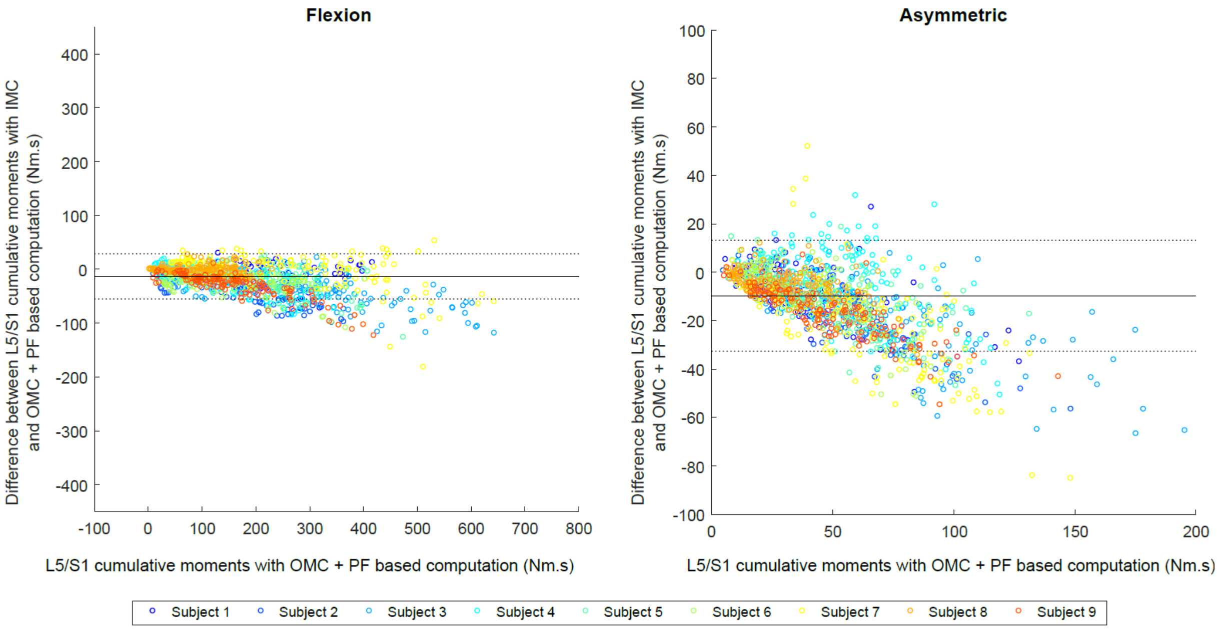

Figure S2. Bland-Altman plot between OMC + PF based and IMC based computations of L5/S1 cumulative moments.
